# Reports of Cases

**Published:** 1898-10

**Authors:** 


### REPORTS OF CASES.

### THREE CASES OF TUMORS, WITH THEIR CLINICAL AND HISTOLOGICAL FEATURES.

By L. Van Es, M.D., V.S.,
MOBILE, ALA.;
VETERINARIAN AND DEMONSTRATOR OF NORMAL AND PATHOLOGICAL HISTOLOGY IN THE MEDICAL COLLEGE OF ALABAMA.

Case I.—The patient, an old mule, came under my observation three years ago. The growth then consisted of a small cystic formation under the skin, situated over the jugular furrow at the lower part of the neck a little above the level of the shoulder-joint. At that time the size of the growth could be materially reduced by pressure, and I suspected that it had some connection with the œsophagus. As the growth did not give rise to any inconvenience, and did not interfere with the usefulness of the animal, I decided to leave it alone.

When I saw the animal again, last summer, the tumor had attained the dimensions of a large cocoanut, and prevented the use of a collar altogether. The skin covering the growth was tense and thin, and at places the hair had entirely disappeared. The tumor did not pulsate, and could not be reduced by pressure, but fluctuated distinctly. The parts below the growth, especially the shoulder and pectoral region, were œdematous, which was probably due to obstruction to the venous circulation. When I inserted a small exploring trocar into the enlargement a few drops of blood appeared, otherwise nothing to explain the fluctuations.

As I was anxious to know the true nature of the contents before attempting any radical operation, I made a small incision and obtained through this a large blood-clot. The removal of the clot was followed by a partial collapse of the enlargement and a profuse hemorrhage, which was stopped with considerable difficulty. The tumor soon regained its former size, and it was evident that I had to deal with some sort of a vascular tumor, and that the only way to get rid of it was to remove it with the knife.

On the following day the animal was secured on the operating-table and, with the necessary precautions as to asepsis, etc., the tumor removed. The skin over the most prominent part, being very thin and adherent to the tumor, an elliptical piece of it was removed with the growth. The base of the neoplasm rested deep into the jugular furrow, and was almost entirely composed of bloodvessels. There were some eight or ten arteries, some of which were the size of a lead-pencil, and a large vein of the thickness of a man's thumb which could plainly be seen to communicate with the jugular vein. After ligating these vessels the tumor was removed with the scissors. The very large wound, treated on the general principles of surgery, healed very rapidly and kindly, the animal making an uneventful recovery.

The tumor, examined after removal, proved to be largely made up of a number of caverns or cavities which communicated freely with each other and with the bloodvessels mentioned before. The cavities were separated from each other by thin septi, which had the appearance of fibrous tissue covered by structures similar to those of the inner lining of normal bloodvessels. All these cavities were distended by one large blood-clot.

In making the microscopical examination I experienced considerable difficulty in finding sections which exhibited any structure by which to judge the true character of the tumor. I stained and mounted a large number of sections, which proved to be in such a state of degeneration as to be entirely devoid of histological elements. I finally succeeded in obtaining some sections which, when properly stained and mounted, showed the following characteristics: Throughout the specimen the fibrous-tissue elements greatly preponderate, while a large number of bloodvessels are to be noticed. Some of these bloodvessels have their walls thickened almost to complete obliteration of the lumen, while others are thin-walled or dilated. In one section the vessel walls appear to be absent, the blood being contained in intercellular canals, strongly suggestive of sarcoma; in these places the cell arrangement somewhat reminds one of sarcoma. In several places the specimen seems to have undergone mucoid degeneration, while in others the process of inflammation is in progress, or an extravasation of red cells is to be noticed. The walls of the large cavities mentioned above do not, as a whole, show any structure themselves, but seem merely composed of the elements of the tumor proper somewhat compressed. In a few places, however, I find the cavity-wall to be made up like a normal vein.

I will not attempt to give this tumor its correct name, as its characteristics are somewhat confusing and its constituents so much degenerated as to greatly prevent a microscopical diagnosis.

Case II.—A large horse, belonging to a logging-camp, was brought to me with the following history:

For considerable time the owner had noticed some difficulty in breathing, which gradually became worse, until, some four weeks before, the horse had become totally unfit for work. In the meantime the owner had discovered the cause of the trouble in the shape of a growth in the nose completely obstructing the passage on the right side. After the owner had made an unsuccessful attempt to remove the growth the animal was placed in my hospital for relief.

On examination I found that a large nasal polypus protruded to within about two inches of the nostril, completely closing the air-passage. By looking into the other nostril it was observed that the nasal septum had bulged considerably toward the left side, and it was only with great effort on the part of the animal that respiration could take place at all. There also was some bulging to be seen externally over the site of the tumor. The animal, of course, also showed the usual symptoms of dyspnœa. It was evident that relief could only be obtained by operative means.

To this end the animal was placed upon the table and an attempt made to remove the tumor by traction, hoping that its attachments would give way. This effort, however, was not successful, and I next made an incision into the nasal cavity, at the angle made by the premaxillary and nasal bones. Inserting my index-finger, I experienced some trouble in forcing my way in, as the growth had crowded into the cavity very tightly. I found the tumor extending up the nasal cavity as far as I could reach; but higher up there was more room, and I could move my finger more freely. After searching some time I discovered the attachment of the growth on one of the turbinated bones, and with a little effort I succeeded in breaking off the piece of bone to which the growth was fastened. The lower end of the polyp was now again grasped with a pair of strong vulsellum forceps and pulled out of the nose without any trouble, as apparently the growth was only attached at the one point spoken of. I now stopped the bleeding by the application of a tampon and released the patient from the table. The following day the tampon was removed and the nose irrigated with some creolin water; this treatment was continued until the surgical wound had healed, which took place in a very short time.

The tumor was about ten inches in length, and must have extended almost into the posterior nares. The weight of it was ten ounces. The attachment to the bone was seen about the middle of the growth. The part below this was hard, dense, and moulded to fit the corresponding part of the nasal cavity. The upper half of the tumor, reddish-white in color, was lobulated and, on account of its soft textures, could be caused to fluctuate. The tumor, which primarily must have developed in the submucous tissue, had a mucous covering and showed evidence of a very rapid growth. As the physical properties of the growths were somewhat different at the various parts, I made sections from the upper, middle, and lower parts of the polypus for microscopical examination.

Sections taken from the upper part of the growth show an abundance of myxomatous tissue, with some fibrous tissue distributed through it, while elastic fibres are also to be noticed. Scattered through the specimens are small nests of epithelial cells in glandular arrangement. Around these alveoli the fibrous tissue appears somewhat denser. Only in one section could bloodvessels be observed. In the middle part there is more fibrous tissue, and the specimen shows a greater vascularity. In some sections the tissue is that of a true adenoma, the alveoli being large and separated by a rather thick fibrous stroma. The elastic fibres present in the upper part of the growth are here entirely wanting. The sections of the lower part of the tumor show, besides the fibrous elements, an abundance of dilated bloodvessels. Through the whole of the sections cellular infiltration is to be seen, while the adenomatous character of the growth above cannot be identified in these parts. I think I am justified in classing this polypus as a myxadenoma.

Case III.—The patient was a small male pony, about two and one-half years old, and was brought to me by the owner, who told me that about six weeks ago the pony's nose had begun to run and that shortly afterward his face had commenced to swell. As at the time the pony had considerable trouble in breathing, and was falling off in flesh, he had thought it best to find some relief for the animal.

On examination I found that the patient had his face enormously enlarged on the left side, so much that he was considerably disfigured. Respiration took place with a great deal of difficulty, accompanied by a loud snoring sound. There was a profuse discharge of muco-pus, and the submaxillary lymph glands were enlarged. The animal was very emaciated, but seemed to be going about very lively.

From the symptoms presented it was evident that I had to deal with a tumor or an accumulation of pus in the maxillary sinus, involving the nasal passage. In either case relief could only be obtained by surgical interference. With this in view, and also for the purpose of setting aside all doubts as to diagnosis, I made an opening into the maxillary sinus by means of a trephine. On inserting my finger I found the sinus to be filled by a new growth; this, of course, excluded the diagnosis of an accumulation of pus and gave the case a more serious aspect. I removed as much of the tumor as possible by means of the sharp spoon, and found that the growth contained a large amount of osseous tissue. The growth had seemingly infiltrated the bony structures forming the antrum Highmorii, as it was very easy to scrape through them with the spoon. As the growth extended into the nasal cavtiy, I also made a trephine opening into the upper part of it, and removed from there a large mass of tumor-tissue, which, however, did not contain anything resembling bone, but appeared to be entirely made up of something resembling fibrous tissue. The tumor extended far into the posterior nares, and, as the patient had now been on the table for some time, I released him and finished the operation the next day, leaving the animal in the standing posture.

The infiltrations into adjacent structures, the rapid growth, the age of the patient, and the presence of bony tissue in the tumor certainly warranted a clinical diagnosis of sarcoma and a most unfavorable prognosis. The after-treatment consisted in daily irrigations with creolin water; the surgical wounds healed very rapidly and the nasal discharge improved, but the cachexia increased, and about three weeks after the operation the animal succumbed in the most miserable condition of emaciation and exhaustion, in spite of the fact that its appetite and other functions remained good until the last.

I did not have opportunity to hold a postmortem, as other duties prevented me from doing so. Sections taken from the soft part of the tumor and stained in lithia-carmine proved it to be a small spindle-celled sarcoma pure and simple. The connective-tissue cells, with large elongated nuclei, forming bundles which intersected each other in different directions, made a beautiful specimen. This part of the tumor is not very vascular; there are, however, some spaces containing blood characteristic of sarcomata. The part of the growths containing bony tissue showed, besides the tissue mentioned in connection with the soft part of the tumor, small plates of bone running parallel to each other and exhibiting something like the Haversian structures seen in normal bone-tissue. I also notice among the spindle-shaped cells some which approach the round form. In connection with this part of the tumor I may mention that there are large spaces containing blood. I could not obtain suitable material to demonstrate microscopically the infiltration of sarcoma-cells into the normal structures.

---

### COMPLICATED MYOCARDITIS.

By Roy E. Jackson, V.S.,
TYLER HILL, PA.

*Subject.* Holstein cow.

*History.* Animal for some time acting unnaturally; loss of spirits; anorexia; milk-secretion almost suppressed.

*Symptoms.* Seen first on November 2, 1897; the animal was much depressed, breathing rather short and quick; respiratory grunt; left flank quite full, and recumbent position assumed most of the time; rumination stopped, eating very little; excretions from bowels scanty and hard. Her temperature was 103° F., pulse 90, full and hard. The jugulars were greatly distended, with distinct jugular regurgitation as high as their bifurcations. Auscultation revealed tumultuous beating of the heart, though the different sounds muffled and indistinct; no friction sounds were audible. Percussion failed to reveal any region where pain was present. No abnormal appearance of the chest walls.

*Diagnosis.* Myocarditis, with impaired action of the third stomach, the former leading to the latter condition and its sequelæ of unthriftiness and anæmic condition. Prognosis doubtful.

*Treatment.* A saline purge was given and every four hours half ounce doses each of nitrate of potash and ginger; no action in twenty-four hours of the purge, a second saline cathartic with one-half ounce of gamboge added, with every four hours one-half ounce doses of ginger with two drachms of powdered nux vomica. Marked flatulence intervening, the trocar and canula were employed, with a few doses of sulphuric ether and dilute alcohol, followed by its subsidence. The following day numerous fluid evacuations of the bowels caused the animal to be brighter, breathing easier, though the respiratory grunt continued when she moved. Appetite better, rumination returned, but was irregular. Powdered gentian root, ginger, bicarbonate of potash, of each three ounces, and capsicum, one ounce, to be given in full tablespoonful doses in one pint of water, were prescribed and the animal turned out in fair weather. Her general condition continued to improve for a time, but, though eating well, she steadily lost in weight. The condition of the heart, jugular pulse, venous distention, continued. On November 18th she was taken with a severe chill, temperature rose to 104° F.; general inappetence. The weather was inclement, and she had remained out, which seemed to account for the chill. A diffusible stimulant was prescribed, and warm clothing. During the night she aborted a seven-months'-old calf. All the digestive symptoms returned, and no relief was obtained by treatment, the cow dying the third day after the second attack.

*Autopsy.* Lungs congested. Heart enlarged to about three times normal dimensions. Pericardium thickened, but closely adherent to the muscular walls. The muscular walls of the right side, extending from the apex to the base, contained a large abscess holding about three quarts of an offensive, thin, light-colored fluid, with numerous yellow inspissated masses of pus in suspension. The left side exhibited two smaller abscesses, toward the median line, entering the ventral septum. The abscess-walls were thin and flabby; several smaller abscesses were also noted, all containing pus. The contents of the third stomach were very dry and hard. I failed to find any specific cause for these extensive changes.

---

### RUPTURE OF THE MESENTERIC ARTERY.

By A. F. Abbott, D.V.S.,
MANCHESTER, N. H.

*Subject.* Gray gelding, about twenty years of age.

*History.* Used for sixteen years in the city fire department in a three-horse hitch. At 10.15. P.M he answered an alarm a block distant, returning at 12.30 A.M., acting in his usual frolicsome manner. At 7.30 o'clock the following morning he winnowed for his breakfast, but was noticed to be ill.

*Symptoms.* Profuse perspiration, wetting his bedding; body cold, while sweat dripped from his abdomen and ran down his legs; trembling of the entire body. No pain was manifested, but his countenance bore an anxious expression; pulse fair.

*Diagnosis.* The animal was placed in a box-stall and a diagnosis of internal rupture made, and a fatal prognosis given.

*Progress.* The perspiration gradually stopped, and in four or five hours he was nearly dry; the nervous trembling almost ceased. The pulse grew gradually weaker and quicker until imperceptible at the jaw. The temperature rose to 105° F.; pain commenced which gradually increased until death at 5.10 o'clock the morning following the attack.

*Autopsy.* On opening the abdominal cavity a small quantity of blood escaped. The peritoneum was more or less congested. Rupture of the mesenteric artery near its origin from the posterior aorta had taken place, with the escape of about three gallons of blood, which had separated the peritoneum from the large colon, and was contained in the pocket thus formed. A cul-de-sac was noted near the point of rupture, the size of one's two fists, its contents partially organized, and which was thought to be the result of a previous small hemorrhage, of which he exhibited no symptoms at the time of its occurrence.

[A closer examination would no doubt have revealed this case to be one due to the parasite strongylus armatus.—Ed.]

---

### ROTATION OR TWIST OF GREAT COLON.

By A. E Williamson, V.S.,
WINNIPEG, MANITOBA.

On the 19th of last June I was called to see the following case:

*Subject.* Gelding, nine years old, weight about 1200 pounds.

*History.* The owner told me the horse had at times been troubled with a stoppage in his urine, and at times showed considerable abdominal pain on being put in the stable at night. He told me he was in the habit of giving the horse potassium nitrate and sweet nitre whenever he saw him showing signs of pain, and in all previous attacks this drench had given relief after the horse had urinated. On this occasion he had administered a drench at night, and on going to the stable in the morning found the horse still showing signs of pain, his bedding all kicked around, and other signs of the horse having been in great pain during the night. I saw the case about ten o'clock in the morning, and found the horse suffering considerable pain from which he seemed to get most relief by lying down stretched out, and he would occasionally lift his head and look back toward his flank.

*Treatment.* I gave a drench composed of spirits ether nitrate, ℥j; ether sulphate, ℥j; chloral hydrate, ℥j; belladonna, fluid extract, ʒij; potassium nitrate, ʒij; aqua, Oj. I passed the catheter and drew off a considerable amount of very dark-colored urine. I gave clysters of warm water several times without much effect, only getting away a few feces in the posterior part of the canal. I repeated the drench about an hour and a half after first drench, but without relieving the symptoms to any extent. About an hour later I administered hypodermically eserine, gr. j; pilocarpine, grs. iij. After waiting for a while and seeing no action from the eserine, I told the owner I thought there was a twist in the great colon. Fifty minutes after injection the horse became very much salivated. I then gave an enema of warm water, but while giving it the horse became partially paralyzed in off hind leg; in a couple of minutes the near hind leg became affected, and he lay down and in a few minutes died almost without a struggle.

*Postmortem.* Examination of the abdominal organs revealed a twist of the great colon at diaphragmatic flexure of colon, anterior to which bowels were full, posteriorly empty. The walls of the bladder were thickened, and it contained a viscid, darkish substance over the mucous membrane.

---

### RECTAL EXAMINATION IN PROTRACTED CASES OF COLIC.[1]

By E. H. Biart, V.S.,
LEAVENWORTH, KAN.

From personal observation and conversations with other veterinarians, I have come to the conclusion that we often neglect to make rectal examination in protracted colics; the failure to do so many times results in the unnecessary sacrifice of the life of our patients. The free colon, floating in the abdominal cavity, usually filled with ingesta, is prone to rotation during violent exercise or rolling attacks of colic, producing a mechanical obstruction which can generally be reduced by manual treatment. Spontaneous reduction I believe to be very exceptional.

I have noted the past few months in nineteen postmortems of cases of colic, six rotations, five to the right and one to the left. Many whom I have conversed with on the subject are disposed to ignore or fail to appreciate the importance of rectal examination.

At 6 P. M. on December 22, 1897, a black mare, seven years old, nervous temperament, was brought to me with a severe attack of spasmodic colic. Antispasmodics and a physic ball were administered, some temporary relief following. At midnight the mare was much worse, with highly congested mucous membranes, small, weak pulse, suppression of abdominal murmurs, and marked tympanites.

[1] Abstract of a report read before the Missouri Valley Veterinary Medical Association.

Enema of the lower bowel was quickly eliminated. Rectal examination revealed a condition that at first indicated an over-distended bladder, but a more complete examination located an empty bladder in the normal position. I realized that this must be a rotation of the colon which required some twenty minutes of manual effort to reduce. Prompt relief followed, with expulsion of gases and solid excreta, the pulse became normal, mucous membranes less congested, and by 2 A.M. the animal was practically well. Failure here to have made a rectal examination would no doubt have proved fatal. I trust others will make observations on these lines and report at our annual meeting, so that we may, from many reports, be better enabled to appreciate the importance of this condition.

---

BACILLI OF BUBONIC PLAGUE.—The recent devastating outbreak of this disease in India, China, and other parts of the Orient has called the attention of bacteriologists to the investigation of the life-history of the bacillus that causes the disease.

"It is said to be a short oval bacillus, usually seen single, sometimes joined end-to-end in pairs, or threes; less commonly as larger threads. It stains more readily at its ends than at its centre. It is sometimes capsulated, is non-sporebearing, is aërobic, and is non-motile. It is found in large numbers in suppurating glands and in much smaller numbers in the circulating blood. It is pathogenic for rats, mice, guinea-pigs, and sheep. The most important source of infection in man is through wounds of the skin."

The anatomical location of the bubonic lesions in the majority of the cases were, according to a recent report of the Municipal Hospital for Infectious Diseases of Bombay, situated in the femoral and femoro-inguinal and axilliary regions of the body. The mortality of the disease was greatest among the poor and ill-fed classes.

---

MISSOURI agricultursists and stock-raisers demand much of the time of State Veterinarian White in lecturing on various subjects of mutual interest at the farmers' institutes throughout the State.

Dr. F. W. Cook, of Hutchinson, Kansas, is one of the pioneer veterinarians of that sunny State, and holds fast to his original location. He is a warm admirer of the trotting horse and handles many clever roadsters as an accessory branch of his profession.

